# Cardiovascular Toxicity of BTKi in Chronic B-Cell Malignancies

**DOI:** 10.31083/RCM47740

**Published:** 2026-06-26

**Authors:** Haojian Gan, Junling Lin, Qiyin Sun, Jiayi Xu, Qingjian He, Shuo Chen, Yu Feng, Bicheng Hu, Wenjuan Wang

**Affiliations:** ^1^School of Medicine, Huzhou University, 313000 Huzhou, Zhejiang, China; ^2^Department of Cardiovascular Center, First affiliated Hospital of Huzhou University, 313000 Huzhou, Zhejiang, China; ^3^Department of Breast and Thyroid Surgery, First affiliated Hospital of Huzhou University, 313000 Huzhou, Zhejiang, China

**Keywords:** Bruton's tyrosine kinase inhibitors, cardiovascular adverse events, atrial fibrillation, drug-related hypertension, hemorrhagic complications, ischemic and hemorrhagic stroke

## Abstract

Bruton's tyrosine kinase (BTK) inhibitors are important targeted drugs for treating chronic B-cell malignancies, and have played a key role in improving patient prognosis and treatment efficacy. However, despite the significant advantages of BTK inhibitors in anti-tumor therapy, the use of these drugs also promotes certain cardiovascular risks, including atrial fibrillation (AF), bleeding, hypertension, heart failure, and potential ventricular arrhythmias (VAs). The latest generation of BTK inhibitors has shown progress in reducing the incidence of these adverse events; however, cardiovascular adverse events (CVAEs) cannot be ignored and continue to pose challenges to patient safety. Therefore, determining how to optimize the clinical application of Bruton’s tyrosine kinase inhibitor (BTKi) further has become an urgent problem. This review systematically analyzes the pathogenesis and clinical manifestations of BTK inhibitor-related cardiovascular complications, with a particular focus on the epidemiological characteristics and potential risk factors of common adverse reactions, such as hypertension, AF, bleeding, and stroke. Moreover, this review explores the pathological and physiological mechanisms, further evaluates the effectiveness of current risk management and intervention strategies, and proposes future research to elucidate mechanisms, optimize doses, and explore combination therapy. Meanwhile, through in-depth analysis and comprehensive evaluation of existing data, this review aims to provide a more scientific basis for clinical decision-making, while promoting research into the safety of continuous BTK inhibitor use to achieve improved treatment outcomes while balancing efficacy and safety.

## 1. Introduction

Bruton's tyrosine kinase (BTK) is a non-receptor cytoplasmic tyrosine kinase produced in B cells and most hematopoietic cells, and is expressed in B lymphocytes, myeloid cells, and platelets [1]. Bruton’s tyrosine kinase inhibitor (BTKi) are used to treat various B-cell malignancies by inhibiting BTK to prevent the proliferation and survival of abnormal B cells. BTK is a key mediator downstream of the B-cell receptor (BCR) signaling pathway, which controls the maturation, survival, and activation of B cells, as well as the production of cytokines and antigen-dependent stimulation of T cells [2]. Research has found that BTKi is not only considered effective for leukemia and lymphoma, but may also have effects on other cell types expressing BTK [3]. However, with the widespread clinical application of BTKi, a number of concerns about its safety have gradually emerged. Clinical data show that although BTKi has achieved significant therapeutic effects in treating various B-cell malignancies, cardiovascular diseases (CVDs) such as hypertension, atrial fibrillation (AF), stuttering, cardiac arrest, and bleeding, have also appeared or worsened in some patients [4]. Cardiovascular toxicity is particularly concerning because these complications not only affect the patients’ quality of life, but may also have a serious negative impact on their overall prognosis. Compared to the first generation of BTKis, the new generation of BTKis, such as acalabrutinib, exhibits increased selectivity in the treatment of various B-cell malignancies and can decrease the risk of atrial arrhythmias. However, the role of acalabrutinib in ventricular arrhythmia (VA) still requires a thorough evaluation [5].

With the expansion of the clinical application of BTKi, its timeliness and safety issues in clinical practice have become increasingly urgent. At present, most studies focus on the applicable population of BTKi and the underlying mechanisms for preventing the occurrence and development of diseases, while attention to its potential cardiovascular toxicity, specific risk factors, and long-term safety management is clearly insufficient [6]. In this analysis, we reviewed the literature and systematically explored the mechanisms and risk factors of the cardiovascular side effects caused by BTKi. Combined with adverse event data in current clinical practice, we have proposed further diagnostic and therapeutic guidelines for improving the safety and management of BTKi. We believe that a deeper understanding of the molecular mechanisms and differences in BTKi-induced cardiovascular toxicity can help develop more precise risk assessments and intervention strategies, thereby achieving more ideal clinical outcomes that balance efficacy and safety. This not only provides a scientific basis for the rational use of BTKi in clinical practice, but also provides direction for optimizing future drug design and treatment plans for promoting the comprehensive, precise, and safe application of BTKi therapy.

## 2. The Incidence of Cardiovascular Toxicity Events Caused by BTKi

There are many problems with BTKi therapy in clinical practice, including its toxicity to the heart, leading to the development or exacerbation of heart-related diseases. Among them, the more common ones include AF, bleeding, hypertension, and stroke [7,8]. Multiple randomized clinical trials, including a study to Evaluate the Efficacy and Safety of Acalabrutinib Versus Ibrutinib in Participants With Relapsed or Refractory Chronic Lymphocytic Leukemia (ELEVATE-RR), A Randomized, Evaluator-Blind, Phase 3 Study of Zanubrutinib Versus Ibrutinib in Patients With Relapsed/Refractory Chronic Lymphocytic Leukemia or Small Lymphocytic Lymphoma (ALPINE), and A Phase 3 Study of Zanubrutinib Versus Ibrutinib in Participants With Waldenström Macroglobulinemia (ASPEN), have demonstrated a stronger correlation between first-generation BTKi treatment and cardiovascular toxicity compared to second-generation BTKi [9,10,11]. AF is the first recognized adverse cardiac reaction of ibrutinib [12]. In several BTKi cardiovascular toxicity studies, AF has been listed as an emergent adverse event [13]. Rapid irregular AF can cause irregular heart rhythms, leading to decreased ventricular function and blood retention in the atria, increasing the risk of thrombosis [14].

In addition to AF, BTKi can also lead to the occurrence or aggravation of hypertension. A study has found that the incidence of hypertension significantly increases among patients receiving BTKi treatment, and some patients’ pre-existing hypertension worsens during treatment [15]. This type of secondary or aggravated hypertension not only affects the cardiovascular status of patients, but may also have adverse effects on the effectiveness of tumor treatment and even lead to treatment interruption or dose adjustment, thereby affecting the overall treatment prognosis.

Hypertension is not only a common cardiovascular complication, but is also an important risk factor for ischemic and hemorrhagic stroke. Continuously elevated blood pressure can significantly increase the shear stress and stress load of the vascular wall, induce vascular endothelial dysfunction, promote the occurrence and progression of atherosclerosis, and further aggravate the risk of thrombosis, thus increasing the risk of stroke [16]. The thrombosis caused by AF, combined with the damage to blood vessels caused by hypertension, further increases the risk of stroke.

Bleeding is also one of the common cardiovascular adverse events (CVAEs) in BTKi treatment [17]. After BTKi treatment for chronic lymphocytic leukemia (CLL) in clinical practice, the risk of bleeding significantly increases. More than half of the patients experience bleeding ranging from mild skin and mucosal bleeding to life-threatening bleeding, most of which are characterized by contusions, nosebleeds, punctate bleeding, hematuria, or ecchymoses Common Terminology Criteria for Adverse Events (CTCAE) grade I–II bleeding. Severe cases may include central nervous system bleeding, such as subdural hematoma or vitreous bleeding. Therefore, it is necessary to weigh the pros and cons during treatment and closely monitor the patient’s coagulation function and potential for bleeding [18,19]. In summary, in order to reduce the incidence of CVAE caused by the clinical use of BTKi and improve patient treatment safety, it is still necessary to understand and evaluate relevant prospective studies to develop better solutions.

However, after systematically retrieving and integrating existing research data, we found that there is significant heterogeneity in the incidence of treatment-related side effects associated with BTKis. Among these, the differences in the types of BTKi are the core factors leading to variations in the incidence of side effects. The baseline heterogeneity of the study population is a key reason for the differences in the incidence of BTKi-related CVAEs [20].

Existing research has confirmed that the prevalence of AF shows a significant upward trend with increasing age [21]. Therefore, if the average age of the study cohort is relatively high, the reported incidence of AF will usually increase accordingly. In addition, patients’ pre-existing underlying CVDs (such as hypertension, diabetes, coronary atherosclerotic heart disease, and heart failure) and history of AF are important risk factors for AF, bleeding events, and stroke during BTKi treatment [21,22,23]. Several studies have shown that patients with a history of CVD have a significantly increased risk of adverse cardiovascular events when receiving BTKi treatment [23]. The incidence of AF, hypertension, bleeding events, and stroke in patients with relapsed/refractory lymphoma may differ from those in patients who undergo initial treatment. The main reason lies in the fact that patients with relapsed/refractory lymphoma are usually in a more critical condition and may have undergone multiple previous treatments, leading to cumulative damage to the cardiovascular system and subsequently increasing the associated risks [24,25]. Patients receiving BTKi treatment often receive concomitant medications (such as chemotherapy drugs), which may themselves have potential cardiovascular toxicity or interact with BTKi, further increasing the risk of cardiovascular events [26]. Furthermore, differences in study designs and diagnostic and monitoring criteria for AF, hypertension, bleeding events, and stroke can also significantly affect the reported incidence rates of related events. Early clinical trials, due to their shorter follow-up periods, may not fully capture the cumulative incidence of AF and late CVAEs [27,28]. The monitoring frequency and detection sensitivity of AF are key factors affecting its rate of detection. Studies that employ continuous electrocardiographic monitoring (such as a dynamic electrocardiogram or an implantable loop recorder) can effectively identify more asymptomatic or paroxysmal AF, thereby increasing the reported incidence of these events. Conversely, relying solely on patient symptom reports or routine electrocardiogram examinations may lead to an underestimation of the true incidence of AF [27,29].

In studies on hypertension, variations in the frequency, timing, and diagnostic criteria for blood pressure measurement (such as the use of ambulatory blood pressure monitoring) can significantly influence the diagnostic rate of hypertension [30]. Additionally, the heterogeneity in reporting bleeding events may arise from differences in the criteria used to define the severity of bleeding and the methods employed for data collection [31]. The diagnostic criteria for stroke, particularly the identification and definition of transient ischemic attacks, can contribute to variations in reported incidence rates [32]. In summary, the differences in the incidence of AF, hypertension, bleeding events, and stroke related to BTKi treatment observed in various studies result from the combined effects of multiple factors discussed earlier. Therefore, when interpreting relevant research data, it is essential to consider the specific type of BTKi, patients’ baseline cardiovascular risk characteristics, study design, monitoring methodologies, and diagnostic criteria for cardiovascular events comprehensively. A thorough analysis and clarification of these influencing factors will aid clinicians in performing individualized cardiovascular risk assessments and precise management for patients undergoing BTKi treatment. This approach will help minimize the risk of adverse cardiovascular events while ensuring the efficacy of BTKi treatment, ultimately optimizing the overall prognosis for patients.

### 2.1 Incidence of AF in BTKi Therapy

The reported incidence of AF is 6% to 16% [23]. In order to further clarify the incidence of AF caused by the use of BTKi, Avalon et al. [33] conducted a retrospective study. They assessed the impact of previous CVD on the development of new AF in patients receiving ibrutinib monotherapy. The median follow-up was 1.1 years. The study concluded that the incidence of AF in CVD patients who had previously received ibrutinib treatment was three times higher than that in CVD patients. The research team also conducted a comparison between BTKi medications and conventional therapeutic agents. They randomly assigned 391 patients with recurrent or refractory CLL or Small Lymphocytic Lymphoma (SLL) to daily treatment with either ibrutinib or the anti-CD20 antibody Ofatumumab. Statistical analysis of the data revealed that 4 patients in the ibrutinib group experienced grade 1 or 2 AF, with 1 patient developing grade 3 or higher AF, leading to the discontinuation of treatment [34]. Wiczer et al. [35] conducted a long-term follow-up of 582 patients receiving ibrutinib treatment, of whom 76 developed AF. The median follow-up was 32 months, and the estimated cumulative incidence rates at 6 months, 1 year, and 2 years were 5.9% (95% CI: 4.2–8.0), 7.5% (95% CI: 5.5–9.9), and 10.3% (95% CI: 8.0–13.0), respectively. The median duration of AF onset in patients was 7.6 months. In a randomized, Open-label Study of Ibrutinib Versus Ofatumumab in Patients With Relapsed or Refractory Chronic Lymphocytic Leukemia or Small Lymphocytic Lymphoma (RESONATE) study comparing ibrutinib and Ofatumumab, the incidence of AF in the ibrutinib group was 10 times higher than that in the Ofatumumab group [36]. Compared to the first-generation BTKi ibrutinib, the newer generation of BTKis, represented by acalabrutinib and zanubrutinib, exhibit significant advantages in cardiac safety, with a significantly reduced risk of cardiac-related adverse events. The ELEVATE-RR study (in patients with previously treated CLL) compared the clinical safety of acalabrutinib and ibrutinib, showing that the incidence of AF in the acalabrutinib group (9%) was significantly lower than that in the ibrutinib group (16%) [7,8]. Another head-to-head study in patients with relapsed/refractory CLL also confirmed that the incidence of AF was lower in the zanubrutinib group compared to the ibrutinib group [9]. The incidence of AF during BTKi treatment is summarized in Table 1 (Ref. [35,37,38,39]).

**Table 1.** **Incidence of AF during BTKi treatment**.

Author	BTKi	Sample of researchers	Experimental design size	Median follow-up time	AF incidence	Type of research
Wiczer TE et al. [35]	Ibrutinib	Hematologic malignancy	582	32 months	13%	Retrospective cohort study
Brown JR et al. [37]	Ibrutinib	CLL/MCL	756	16.6 months	6.5%	Randomized controlled trial
Byrd JC et al. [38]	Acalabrutinib	CLL patients previously treated for del(17p) or del(11q)	268	40.9 months	9.40%	Multicenter randomized phase III clinical trial
Visentin A et al. [39]	Ibrutinib	CLL	354	25 months	12%	Retrospective cohort study

CLL, Chronic Lymphocytic Leukemia; MCL, Mantle Cell Lymphoma; AF, atrial fibrillation; BTKi, Bruton’s Tyrosine Kinase inhibitor.

### 2.2 Incidence of Hypertension in BTKi Therapy

In existing studies on hypertension caused by BTKi treatment, the incidence of hypertension is variable and is most likely due to various types of anti-hypertensive drugs that were used to treat these patients [15]. A study from the Ohio State University Medical Center showed that among 280 patients treated with acalabrutinib, 48.9% developed new/worsening hypertension within 41 months, and the cumulative incidence of new hypertension after 1 year was 53.9%, of which 1.7% were ≥ grade 3 hypertension [15]. In a study on the incidence of ibrutinib-related hypertension, it was observed that more than 75% of ibrutinib users had new or worsening hypertension within 30 months in 562 consecutive cases of patients receiving ibrutinib for the treatment of B-cell malignant tumors. New episodes of hypertension occurred in 71.6% of ibrutinib users, and reached 50% of the cumulative incidence within 4.2 months. Among patients without previous hypertension, 17.7% developed > grade 3 hypertension [40]. These data indicate that BTKi with acalabrutinib and ibrutinib increase both the incidence and severity of hypertension. A large population of B-cell cancer patients who received continuous treatment with acalabrutinib was evaluated for the efficacy of standard antihypertensive drugs in preventing hypertension induced by acalabrutinib and ibrutinib. The results showed that no single antihypertensive drug could prevent hypertension related to acalabrutinib and ibrutinib [15,40]. Caldeira et al. [41] selected 8 randomized controlled trials to compare ibrutinib with a control group (placebo, no treatment, or standard care, non-pharmacological intervention, or any active medication), with a total of 2580 patients (54.7% receiving ibrutinib treatment). They demonstrated that ibrutinib was significantly associated with an increased risk of hypertension, with a hazard ratio of 2.82 (95% CI: 1.52–5.23) and moderate quality of evidence [41]. Table 2 (Ref. [42,43,44]) presents the incidence of hypertension reported in key clinical studies of BTKi therapy. Given the rapid increase in the number of BTKi users and the lack of effective tools or biomarkers to determine the risk of hypertension, these observations are crucial. In summary, these research results indicate that BTKi can increase the incidence of hypertension and its management during the treatment of lymphocytic tumors and cancers.

**Table 2.** **Incidence of hypertension during BTKi treatment**.

Author	BTKi	Sample of researchers	Experimental design size	Median follow-up time	Incidence of hypertension	Type of research
Byrd JC et al. [42]	Ibrutinib	CLL/SLL (Treatment-Naive)	31	30 months	23%	clinical follow-up study
Byrd JC et al. [42]	Ibrutinib	CLL/SLL (Relapsed/Refractory)	101	23 months	20%	Phase 1b/2 study
Byrd JC et al. [43]	Ibrutinib	CLL	265	35.5 months	23.2%	clinical follow-up study
Burger JA et al. [44]	Ibrutinib	CLL/SLL (Treatment-Naive)	136	9.6 years	30%	Randomized Phase 3 Trial

CLL, Chronic Lymphocytic Leukemia; SLL, Small Lymphocytic Lymphoma.

### 2.3 Incidence of Bleeding Events in BTKi Therapy

There is no fixed range for the incidence of bleeding during BTKi treatment, which varies depending on the specific drug, the patient’s baseline conditions, and concomitant medications. The overall incidence ranges from 22% to 50%, and most cases are mild to moderate [1]. In 2018, Jennifer R et al. [45] analyzed the incidence and risk factors of major bleeding in 15 clinical studies with ibrutinib. They reported that 39% of patients taking ibrutinib experienced any level of bleeding. Jan et al. [46] also conducted a related survey, randomly assigning 269 patients aged 65 years and above (median age of 73 years) with CLL or small lymphocytic lymphoma, who had not received prior treatment, to be treated with ibrutinib and Chlorambucil. Long-term follow-up found that there were 4 patients with grade III bleeding and 1 patient with grade IV bleeding in the ibrutinib group. The research team also conducted a literature review and analysis of previous reports on BTKi bleeding events, and found that the combined annual bleeding incidence of BTKi was 20.8/100 patients/year (95% CI: 19.1–22.1). The relative risk of any bleeding combined with ibrutinib was 2.72 (95% CI: 1.62–4.58; *p* = 0.0002) [47]. Table 3 (Ref. [34,48,49]) summarizes the bleeding incidence rates reported in key clinical trials of BTKi therapy. Acalabrutinib is a BTKi second-generation drug with higher selectivity, which was approved by the Food and Drug Administration (FDA) for clinical treatment in 2017 [50]. To investigate the bleeding caused by this new BTKi, researchers conducted a phase 1–2 trial of acalabrutinib treatment on 61 patients with recurrent CLL. They found that mild adverse events, including bruising and contusions, were common during the patient treatment process, but no major bleeding events had occurred [51]. An ASPEN Phase III clinical trial (NCT03053440), evaluated the safety differences between two generations of BTKi by comparing the adverse reactions of patients with relapsed/refractory Waldenström macroglobulinemia (WM) treated with zanubrutinib and ibrutinib. The results showed that the incidence of minor bleeding (49%) and severe bleeding (6%) in the zanubrutinib group was lower than that in the ibrutinib group (59% and 9%, respectively). Therefore, in terms of bleeding-related safety, the new generation BTKi drug zanubrutinib is superior to ibrutinib [52]. An analysis of the literature indicates that the bleeding rate associated with BTKi treatment is relatively high. Currently, there is almost no information available to guide physicians in making decisions on antiplatelet therapy while using BTKi to treat patients [18,47,53]. There is still a great potential risk for patients receiving treatment with this type of medication, and it is urgent to develop an alternative BTKi or a lower-risk BTKi.

**Table 3.** **Incidence of bleeding during BTKi treatment**.

Author	BTKi	Sample of researchers	Experimental design size	Median follow-up time	Incidence of bleeding	Type of research
Hillmen P et al. [48]	Zanubrutinib	CLL/SLL (Relapsed/Refractory)	204	15.3 months	35.8%	Phase III trial
Hillmen P et al. [48]	Ibrutinib	CLL/SLL (Relapsed/Refractory)	207	14.6 months	36.2%	Phase III trial
Wang ML et al. [49]	Ibrutinib	MCL	111	6 months	41%	Phase 2 registration trial
Byrd JC et al. [34]	Ibrutinib	CLL/SLL	195	8.6 months	44%	Phase 3 trial

CLL, Chronic Lymphocytic Leukemia; SLL, Small Lymphocytic Lymphoma; MCL, Mantle Cell Lymphoma.

### 2.4 Incidence of Stroke in BTKi Therapy

The incidence rate of stroke caused by BTKi treatment varies and is due to several factors, such as the drug type, the patient’s underlying diseases, treatment regimens, and the duration of treatment [4]. A study analyzed 582 patients who received ibrutinib treatment and found that only one case of stroke-related symptoms occurred in patients with AF treated with ibrutinib therapy [35]. Similarly, in a study based on a disproportionality analysis of adverse drug reactions reported by the WHO Global Individual Case Safety Report Database (VigiBase), ischemic stroke was a rare complication, and approximately 20% of stroke events in the study were reported in conjunction with supraventricular arrhythmias. However, there is little data on differences in onset within the same case [52]. Table 4 (Ref. [54,55]) presents the incidence of stroke reported in key clinical studies of BTKi therapy. However, in a recent study comparing the incidence of stroke, AF, myocardial infarction, and bleeding in CLL patients treated with and without ibrutinib in the SEER-Medicare database, patients treated with ibrutinib had a 1.91-fold increased risk of stroke [8].

**Table 4.** **Incidence of stroke during BTKi treatment**.

Author	BTKi	Sample of researchers	Experimental design size	Median follow-up time	Incidence of stroke	Type of research
Ammad Ud Din M et al. [55]	Ibrutinib	CLL with AF	7265	not mentioned	3.09%	Retrospective cohort study
Ammad Ud Din M et al. [55]	Ibrutinib	CLL without AF	7265	not mentioned	1.65%	Retrospective cohort study
Nunes RAB et al. [54]	Acalabrutinib	CLL/SLL	2107	3 years	2.5%	Retrospective cohort study
Nunes RAB et al. [54]	Ibrutinib	CLL/SLL	2107	3 years	2.7%	Retrospective cohort study

CLL, Chronic Lymphocytic Leukemia; SLL, Small Lymphocytic Lymphoma.

## 3. Mechanisms Related to BTKi-Induced Cardiovascular Toxicity Events

### 3.1 PI3K/Akt Pathway Inhibition

The phosphoinositide 3-kinase (PI3K)-Akt pathway [56] is one of the pathways regulated by BTK and Tyrosine-protein kinase Tec (TEC), which plays a critical role in cell growth, survival, and metabolism. Its p110α subtype is critical for cardiac protection [57]. Qin et al. [58] studied HL-1 cells treated with ibrutinib and found that BTKi promotes autophagy in cardiomyocytes by inhibiting the PI3K-AKT-mTOR signaling pathway, leading to the degradation of connexins, which disrupts the electrical coupling between cardiomyocytes and promotes the occurrence of AF. BTKi may also directly or indirectly affect cardiac electrophysiology and structural remodeling by inhibiting the PI3K/Akt signaling pathway, thereby increasing the risk of AF [59]. In this pathway, the alpha subunit of PI3K is the target of ibrutinib. Ibrutinib, acting on the alpha subunit, may inhibit the phosphorylation of Akt after Insulin-like growth factor 1 receptor (IGF1R) stimulation of PI3K, weaken the inhibitory effect of Akt on fibroblasts, and thus cause atrial fibrosis, increasing the risk of AF [13]. In terms of the cardiovascular effects related to the PI3K/Akt pathway, the interference of zanubrutinib and acalabrutinib with this pathway is significantly weaker than that of ibrutinib. For AF, these two drugs have a weaker inhibitory effect on the PI3Kα subunit, which can reduce the inhibition of the PI3K-AKT-mTOR signaling pathway, thereby alleviating cardiomyocyte autophagy, decreasing the degradation of connexins, and reducing the disruption of electrical coupling between cardiomyocytes [60].

Inhibition of PI3K can also significantly enhance the late sodium current INa and Late, and prolong the duration of the action potential, which affects the normal electrophysiological characteristics of the heart and increases the risk of AF [61]. BTKi may also cause vasoconstriction through the PI3K/Akt pathway, leading to hypertension. The activation of the PI3K/Akt pathway can promote the synthesis and release of nitric oxide (NO) by phosphorylating endothelial nitric oxide synthase (eNOS), which plays a central role in maintaining endothelial function [62]. BTKi may impair endothelial-dependent vasodilation and enhance vascular smooth muscle contraction by inhibiting this pathway.

### 3.2 Endothelial Dysfunction

BTKi may increase the risk of AF by affecting endothelial cell function, leading to decreased vasodilation and altered hemodynamics [60]. Endothelial cells play a crucial role in maintaining vascular homeostasis, and their dysfunction can lead to an increase in the inflammatory response and oxidative stress, which are potential triggers of AF [63]. The use of BTKi can lead to an increase in the generation of reactive oxygen species (ROS), which can cause damage to endothelial cells and affect their normal function, including decreased vasodilation and impaired integrity of the endothelial barrier [64,65]. Oxidative stress can also induce endothelial cell apoptosis, further impairing cell function. A study compared the protein expression of mice treated with ibrutinib with that of normal mice. The results showed that the expression of ROS-related proteins, including nicotinamide adenine dinucleotide phosphate (NADPH) oxidase 2 (NOX2), NADPH oxidase 4 (NOX4), p22 phox, xanthine oxidase (XO), and transforming growth factor-beta (TGF-β), was significantly increased in the ibrutinib group. Additionally, the expression of oxidized calcium/calmodulin-dependent protein kinase II (ox CaMKII), phosphorylated CaMKII (p-CaMKII) (Thr-286), and phosphorylated ryanodine receptor 2 (p-RyR2) (Ser2814) was also significantly increased, leading to abnormal sarcoplasmic reticulum Ca^2+^ release and enhanced mitochondrial structure. These findings helped to clarify the pathophysiological role of ROS signaling in atrial and ventricular myocardium, providing new insights for future studies on the mechanism of ibrutinib-induced AF [66]. The reduction of NO synthesis and the inflammatory response of endothelial cells can also affect the normal function of endothelial cells. BTKi may increase the incidence of hypertension through this mechanism. A study found that dysregulation of eNOS can lead to a decrease in NO bioavailability, which in turn can cause endothelial dysfunction and hypertension [67]. The inflammatory response of endothelial cells increases the adhesion between white blood cells and endothelial cells, further impairing vascular function and leading to an increase in vascular tension and elevated blood pressure [68]. In addition, Endothelin-1 (ET-1) is a potent vasoconstrictor peptide produced by endothelial cells. By binding to Endothelin Receptor A (ETA) and Endothelin Receptor B (ETB), it triggers the strong contraction of vascular smooth muscle, leading to an increase in blood pressure [69]. Therefore, BTKi may also enhance the ET-1 signaling pathway, further exacerbating vascular constriction and leading to hypertension. Ibrutinib damages vascular integrity and increases the risk of adverse events by inducing the Bone Morphogenetic Protein 4 (BMP4) protein [70]. In 2014, Tsuchida et al. [71] treated human microvascular endothelial cells with BMP4 protein and compared them with those without BMP4 protein, thus confirming the role of BMP4 protein in promoting angiogenesis. Subsequent research found that ibrutinib can also induce endothelial dysfunction by upregulating BMP4 expression, thereby significantly inhibiting the ability of lumen formation by Human Umbilical Vein Endothelial Cells (HUVECs). In contrast, zanubrutinib has a weaker activating effect on BMP4 and inhibitory effect on lumen formation of HUVECs, while acalabrutinib has almost no such effect [72]. To explore whether BTKi have similar inhibitory effects on normal blood vessels, researchers pretreated HUVECs with various BTKi and then seeded them on Matrigel matrices. Through a series of experiments, they found that the levels of BMP4 and THBS1 in HUVECs treated with ibrutinib were significantly higher than those in the group treated with zanubrutinib, and that low concentrations of ibrutinib could effectively disrupt vascular integrity. In addition to ibrutinib, other BTKi require high concentrations to damage or even leave the blood vessels intact. The research team also conducted Western blot analysis at the protein level and *in vitro* angiogenesis experiments. The Western blot results showed no expression of BTK in HUVECs, indicating that BTKi regulates BMP4 expression through a BTK-dependent pathway. The absence of BTK expression in cells, combined with *in vitro* angiogenesis results, showed that ibrutinib can disrupt vascular integrity when the concentration of rhBMP4 protein is 200 ng/mL [72].

BTK participates in Toll-like receptor (TLR) and chemokine signaling. This inhibition may disrupt immune balance, leading to abnormal release of inflammatory factors and affecting the stability of the vascular endothelium [73]. BTKi can also reduce the production of pro-inflammatory cytokines such as Tumor Necrosis Factor-α (TNF-α), Interleukin-1β (IL-1β), and Interleukin-6 (IL-6) by inhibiting BTK activity, thereby reducing the inflammatory response. This inhibitory effect may also lead to a weakened protective function of immune cells against the vascular endothelium, thereby increasing the risk of bleeding.

BTKi may also result in strokes by causing endothelial dysfunction. BTKi may also lead to endothelial dysfunction by inhibiting eNOS activity and reducing NO production. When endothelial dysfunction occurs, tissue-type plasminogen activator (t-PA) secreted by endothelial cells decreases, while plasminogen activator inhibitor-1 (PAI-1) increases, resulting in a decrease in fibrinolytic system activity and a hypercoagulable state [74].

After an endothelial cell injury, collagen fibers under the endothelium are exposed, activating coagulation factor XII, initiating endogenous coagulation pathways, and further promoting thrombus formation. If blood vessels in the brain form thrombi or if thrombi detach and block the blood, it can lead to a stroke [72].

Due to the bidirectional effects of BTKi on the coagulation system, different pathogenic mechanisms and underlying patient co-morbidities may lead to bleeding or thrombosis. In summary, the mechanism of BTKi-related stroke is complex and is multi-factorial. Current research suggests that the stroke mechanism involves both hypercoagulable factors and coagulation mechanisms, which work together to cause diverse clinical manifestations and increase the difficulty of making a clinical diagnosis.

### 3.3 Off Target

Although the new generation of BTKi (such as acalabrutinib and zanubrutinib) has higher selectivity for BTK, there are still some off-target effects. These off-target effects are often considered to be the cause of some of the adverse reactions induced by ibrutinib [23]. Off-target kinase inhibition is considered an important reason for the occurrence of AF. BTKi not only inhibits BTK targets, but also suppresses them by inhibiting several tyrosine kinases and their pathways, such as Epidermal Growth Factor Receptor (EGFR), Interleukin-2-Inducible T-cell Kinase (ITK), and Tec Protein Kinase (TXK), thereby exhibiting off-target activity [23]. BTK and TEC are expressed in cardiac tissue and have higher concentrations in atrial tissue [75]. Alexandre et al. [75] obtained publicly available microarray data from the National Center for Biotechnology Information (NCBI)’s gene expression database to evaluate gene expression in human heart tissue. Through a series of data analyses, it was found that under AF conditions, the expression levels of BTK and TEC transcripts in human heart tissue were significantly higher than those in sinus rhythm, indicating that BTK and TEC may have functional roles under conditions of cardiac stress and their inhibition (such as through the action of ibrutinib) may lead to the occurrence of AF. The EGFR signaling pathway also plays a key regulatory role in cardiac fibroblasts. Inhibition of EGFR can affect the proliferation of fibroblasts and the synthesis of extracellular matrix [76]. In addition, studies have shown that sustained high concentrations of ibrutinib in the blood significantly inhibit the expression of C-terminal Src kinase (CSK). CSK is an endogenous inhibitor or brake of Src family tyrosine kinases (SFK) [77]. After knocking out specific CSK in mouse hearts, Xiao et al. [77] found an increase in AF, left atrial enlargement, fibrosis, and inflammation in mice. Therefore, it can be concluded that higher drug concentrations in the blood of patients treated with ibrutinib inhibit the expression of CSK, leading to AF, but the specific mechanism is still unclear. BTKi off-target inhibition of Src family kinase CSK can lead to AF, thereby increasing the risk of stroke [78].

Bleeding is also closely related to the off-target effects of BTKi. When TEC kinases are inhibited, platelets cannot normally aggregate, thereby increasing the risk of bleeding, which may further lead to hemorrhagic stroke [79,80]. Ibrutinib also inhibits Src kinases, which affect platelet adhesion to collagen, thereby increasing the tendency for bleeding [81]. Compared to first-generation BTKi, second-generation BTKi, represented by acalabrutinib and zanubrutinib, significantly enhances selectivity towards the BTK target. Acalabrutinib can achieve irreversible inhibition of BTK by forming a covalent bond with the cysteine residue (Cys481) at the active site of BTK, thereby inhibiting downstream target proteins while reducing the inhibitory effect on non-BTK targets [23,82]. Zanubrutinib is also designed to more efficiently select the BTK target, reducing the side effects caused by binding to non-BTK targets and lowering the risk of complications [83,84]. *In vitro* genomic analysis showed that zanubrutinib has significantly higher selectivity for BTK than ibrutinib and acalabrutinib [83].

### 3.4 Platelet Dysfunction

Previous studies have suggested that the increased bleeding rate of ibrutinib is caused by platelet dysfunction [85,86]. It has been confirmed that BTKi can inhibit the downstream regulation of phospholipase Cγ2 (PLCγ2) in the platelet collagen receptor glycoprotein (GPVI) by inhibiting TEC, a member of the tyrosine kinase family. This results in the inability of platelet signaling to initiate conduction, platelet activation, and coagulation. GPVI is responsible for platelet activation and aggregation in response to exposure to collagen and collagen-related peptides [79,80,81]. However, the inhibitory effects of different BTKi on GPVI vary greatly. The inhibitory effects of zanubrutinib and acalabrutinib on GPVI-mediated platelet aggregation are weaker than those of ibrutinib. Therefore, compared to second-generation BTKi, they have a smaller regulatory impact on GPVI downstream phospholipase PLCγ2, resulting in a milder inhibition of platelet signaling, which preserves the activation and coagulation function of platelets [87].

Barf et al. [82] have demonstrated that ibrutinib selectively inhibits platelet signaling and function downstream of GPVI, and strongly affects the firm adhesion of platelets to von Willebrand factor (vWF) during arterial blood flow. A study found that when the vascular hemophilia factor vWF of the Glycoprotein Ib (GPIb) platelet receptor linked to tyrosine kinase is inhibited by BTKi, GPIb cannot mediate platelet collagen adhesion through vWF, increasing the risk of bleeding [79]. The study also randomly divided CLL patients and normal patients into a healthy control group, an untreated patient group, a zanubrutinib treatment group, and an ibrutinib treatment group. They integrated and compared the changes in the content of integrin αIIbβ3 on platelets in each group of patients, and found that the expression of integrin αIIbβ3 on the platelets of CLL patients treated with ibrutinib was significantly reduced [86]. In addition, in experimental studies, Mendez-Ruiz et al. [79] demonstrated that ibrutinib induces the shedding of integrin αIIbβ3, which affects platelet adhesion, aggregation, and signaling functions, leading to inhibition of platelet activation and further weakening of platelet clot retraction and stability.

BTKi may inhibit the activity of factor XIIa, resulting in abnormal coagulation cascade reactions, which impair coagulation function. When cerebral vascular disease occurs, the coagulation mechanism cannot be activated to perform reparative processes. Therefore, blood clots cannot be effectively formed, which may lead to aneurysm rupture and bleeding or local brain tissue infarction due to ischemia, ultimately causing a stroke [83].

### 3.5 Activation of the Sympathetic Nervous System

Overactivation of the sympathetic nervous system (SNS) can increase cardiac output and peripheral vascular resistance, leading to hypertension [84]. BTK is expressed in the central nervous system, and BTKi may interfere with normal neural regulation by inhibiting BTK in the central nervous system, leading to increased sympathetic nervous activity [88]. BTKi may indirectly enhance SNS activity by affecting the regulation of the Renin-Angiotensin-Aldosterone System (RAAS). There is a close interaction between RAAS and the SNS in blood pressure regulation.

The activation of the SNS is also associated with the occurrence of BTKi-related stroke. BTKi may stimulate the activity of SNS by inducing an inflammatory response and releasing inflammatory factors IL-6 and TNF-α. Overactivation of the SNS leads to increased blood pressure, faster heart rate, and vasoconstriction, which may increase the risk of stroke [89]. The activation of the SNS is also associated with cardiac fibrosis and inflammation. Zanubrutinib, due to its higher selectivity for BTK, exhibits significant advantages in reducing cardiac fibrosis and inflammation caused by neural activation [90].

### 3.6 Vascular Reconstruction

Vascular remodeling is one of the pathological features of hypertension. The abnormal proliferation and migration of Vascular Smooth Muscle Cells (VSMCs) are key steps in vascular remodeling, which may lead to thickening of the vascular wall and increased vascular resistance, leading to changes in vascular structure, thereby causing hypertension [91]. It may lead to vascular remodeling by affecting the proliferation and migration of VSMCs. In addition, inflammation can also affect vascular remodeling. BTKi may regulate the expression of inflammatory factors such as TNF-α and IL-6 by inhibiting BTK, reducing macrophage activation and polarization, and affecting the structure and function of vascular walls, leading to hypertension [26,92]. BTKi may also affect the expression of Extracellular Matrix (ECM) related genes and the balance between ECM synthesis and degradation. BTKi may promote the synthesis of more ECM components, such as collagen and fibronectin, in fibroblasts, while inhibiting the activity of degradation enzymes, such as matrix metalloproteinases, leading to excessive deposition of ECM in the vascular wall. The remodeling of the ECM can cause the vascular wall to become stiffer and less elastic, further exacerbating vascular stenosis and hemodynamic abnormalities, which can lead to the formation of blood clots that can enter the cerebral blood vessels and cause a stroke [87].

### 3.7 Abnormal Calcium (Ca^2+^) Ion Channels

**Fig. 1.**
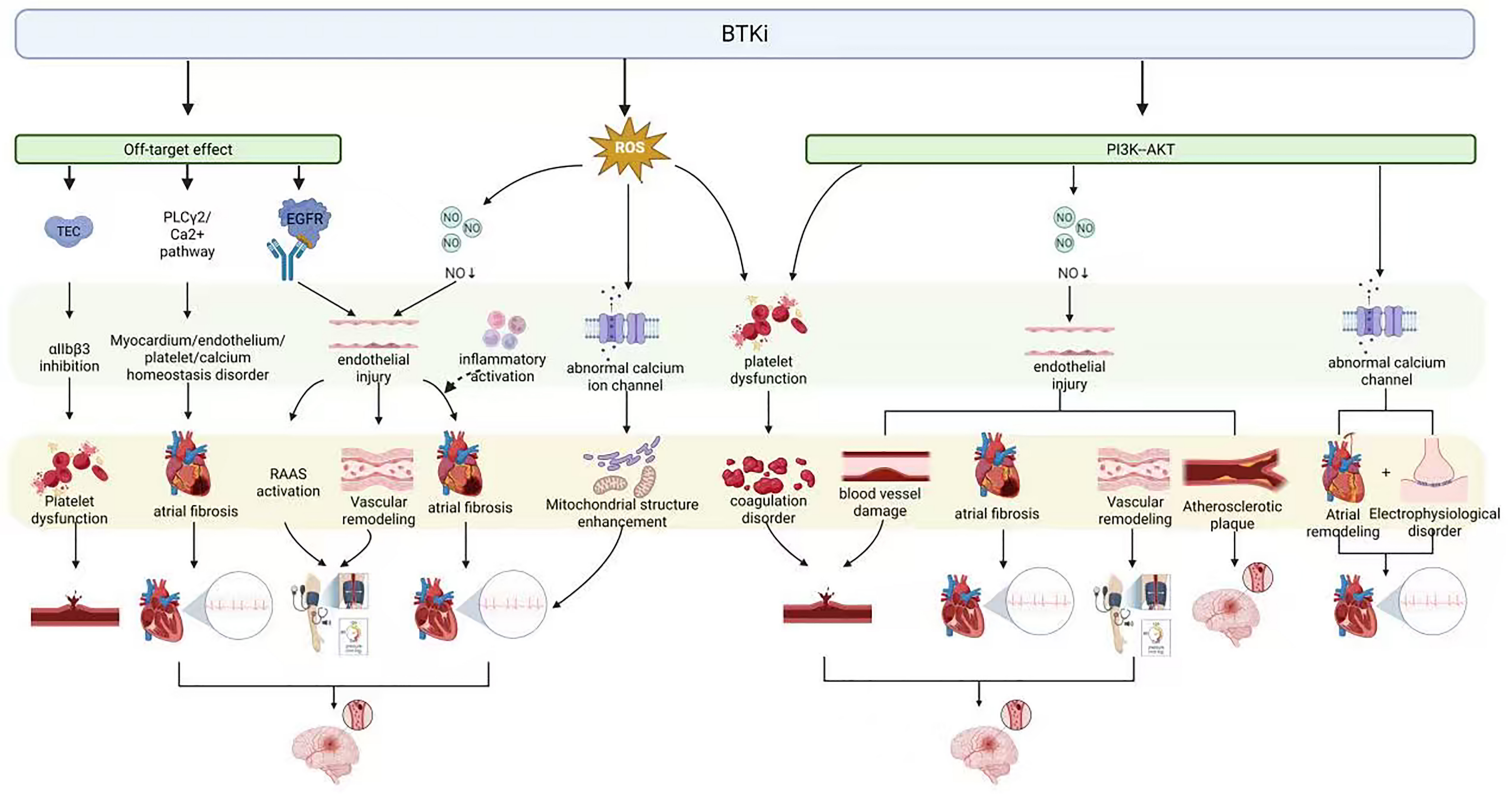
**Four-level progressive mechanism diagram of BTKi-related clinical events**. The core mechanism of BTKi-induced cardiovascular toxicity lies in the abnormalities in signaling pathways such as PI3K/Akt inhibition, elevated ROS, and off-target effects. These changes progress step by step through three intertwined core pathways: off-target effects, ROS release, and PI3K-Akt activation, which are interrelated and synergistic. Initially, they trigger cellular dysfunction, subsequently induce structural and functional abnormalities at the organ or systemic level, and ultimately jointly contribute to BTKi-related cardiovascular toxicity events such as bleeding, stroke, AF, and hypertension. The black arrow pointing down means decrease. This image was created by https://BioRender.com (accessed on October 20, 2025). EGFR, Epidermal Growth Factor Receptor; TEC, Tec Kinase; RAAS, Renin-Angiotensin-Aldosterone System; ROS, reactive oxygen species.

**Fig. 2.**
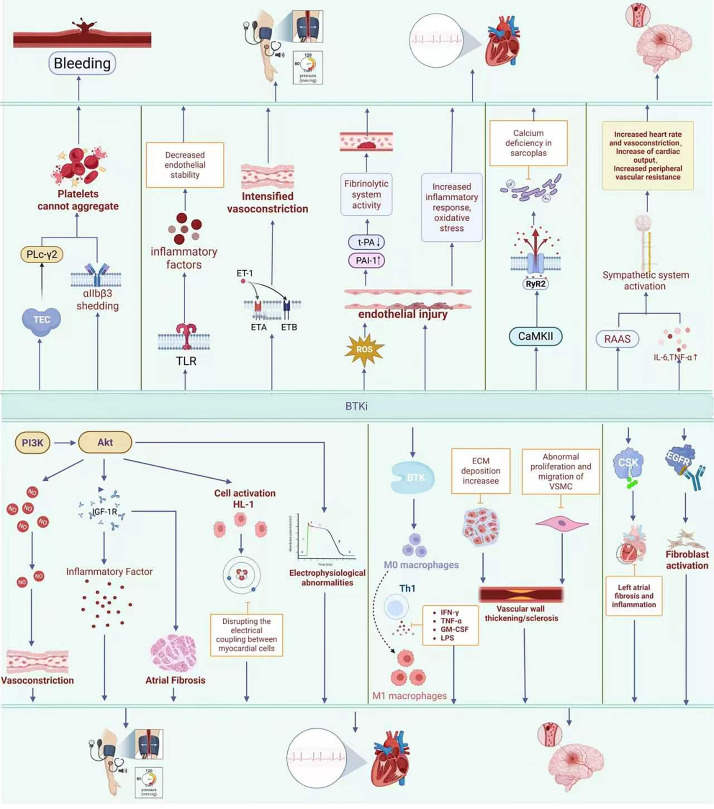
**Multiple regulatory mechanisms of BTKi-induced cardiovascular toxicity events**. The mechanisms by which BTKi triggers cardiovascular events in clinical practice can be divided into two major categories: primary and secondary, involving a total of seven specific pathways. The primary mechanisms include four core pathways: off-target effects, endothelial dysfunction, inhibition of the PI3K/Akt pathway, and platelet dysfunction. In addition to the above primary mechanisms, there are three additional pathways that can be classified as secondary mechanisms: sympathetic activation, vascular remodeling, and calcium channel abnormalities. The downward arrow means decreases, and the upward arrow means increases. This image was created by https://BioRender.com (accessed on October 20, 2025). BTKi, Bruton’s Tyrosine Kinase inhibitor; PI3K, Phosphoinositide 3-kinase; ECM, Extracellular Matrix; VSMC, Vascular Smooth Muscle Cell; CSK, C-terminal Src Kinase; EGFR, Epidermal Growth Factor Receptor; TEC, Tec Kinase; TLR, Toll-Like Receptor; ETA, Endothelin Receptor Type A; ETB, Endothelin Receptor Type B; ROS, Reactive Oxygen Species; CaMKII, Calcium/Calmodulin-Dependent Protein Kinase II; RyR2, Ryanodine Receptor 2; RAAS, Renin-Angiotensin-Aldosterone System.

The mechanism of BTKi-induced AF may also be related to calcium channel abnormalities. Studies have found that AF mice induced by four weeks of ibrutinib administration showed a decrease in calcium transient amplitude, increased spontaneous calcium release, decreased sarcoplasmic reticulum calcium storage capacity, and calcium processing disorders associated with increased calcium/calmodulin-dependent protein kinase II (CaMKII) activity. The overactive RyR2 channel is crucial for inducing and maintaining AF, and RyR2-S2814 is a key phosphorylation target of CaMKII. This phosphorylation process is crucial in regulating intracellular calcium homeostasis. Therefore, ibrutinib treatment may enhance CaMKII activity, causing excessive opening of RyR2 and leading to Ca^2+^ leakage in SR, thereby triggering abnormal intracellular Ca^2+^ transients and inducing AF [93]. Fig. 1 illustrates the multi-stage mechanism of BTKi-related cardiovascular adverse events. The detailed mechanism of BTKI-induced cardiovascular disease is shown in Fig. 2.

## 4. Clinical Management of BTKi-Related Cardiovascular Toxicity Events

AF is a frequent adverse cardiovascular event in patients with CLL undergoing BTKi therapy [94,95,96]. In clinical practice, AF is usually controllable but requires lifelong treatment. Symptoms of AF may include palpitations, dizziness, fainting, fatigue, heart failure, or thromboembolism. Severe cases may result in stroke, but it may also be asymptomatic. Its main complications include heart failure and arterial thromboembolism leading to stroke or other acute ischemic syndromes [13]. AF not only results in a deterioration of patients’ quality of life but also elevates the risk of stroke fivefold and the risk of mortality twofold [66]. At present, there is no optimal management for BTKi-related cardiac adverse events in clinical practice [23]. Second-generation BTKi, which exhibit lower cardiovascular toxicity compared to ibrutinib, are recommended for high-risk cardiovascular patients [90,97]. Studies suggest that cardiac events should be treated and managed in a multidisciplinary team composed of cardiac oncologists and hematologists [98]. Throughout the team-based treatment process, hematologists are tasked with conducting comprehensive evaluations of CLL patients, initiating BTKi therapy, and monitoring treatment responses as well as potential risks [94]. Cardio-oncologists are invaluable members of this team. Should any warning signs emerge during the initial examination, particularly AF on the baseline electrocardiogram, it may be necessary to seek consultation from a cardio-oncologist and promptly adjust the drug type and dosage [94,99]. Therefore, prior to administering BTKi to patients, a comprehensive assessment of multiple patient-related factors is essential to devise a personalized treatment plan. In addition, regular electrocardiograms and dynamic electrocardiographic monitoring should be conducted during treatment to minimize the risk of new-onset or recurrent AF in patients [23,94,95].

Hypertension is a risk factor for AF, so closer monitoring and comprehensive evaluation are needed to determine the risk of hypertension. For patients with hypertension and AF comorbidities, the risk of stroke is considered to be increased [100]. Therefore, prior to initiating BTKi therapy for these patients, it is essential to evaluate their stroke risk using tools such as the Congestive heart failure, Hypertension, Age ≥75 years (doubled), Diabetes mellitus, Stroke/TIA/thromboembolism (doubled), Vascular disease, Age 65–74 years, Sex category (female) (CHA_2_DS_2_-VASc) score and taking into account their history of CVD [94,101]. This evaluation aids in formulating a personalized treatment plan with relatively low risk. For patients at stroke risk, anticoagulant therapy should be considered in accordance with scientific guidelines [100]. Patients are also advised to implement lifestyle modifications, including a low-salt diet, moderate physical activity, smoking cessation, and limited alcohol intake, to manage hypertension and mitigate the associated risk of stroke [94]. Throughout the treatment, close monitoring of the patient’s cardiovascular status is imperative, encompassing regular blood pressure checks, electrocardiograms, monitoring of symptoms, and routine laboratory tests. The types of medication and their dosage should be adjusted promptly based on the patient’s response, and antihypertensive drugs should be introduced as necessary to maintain blood pressure control [90,94,102]. Once a stroke is detected, BTKi should be discontinued immediately, and the patient should be promptly transported to a hospital equipped to handle strokes [94,103]. At present, there are no recommended drugs for the treatment of BTKi-related hypertension. However, research has shown that the incidence of hypertension is lower with second-generation BTKi (such as acalabrutinib). Therefore, for patients at a higher risk of hypertension, the use of second-generation BTKi medications may be considered [15]. Studies indicate that managing hypertension also necessitates collaboration among hematologists, cardiologists, and nephrologists. Hematologists are responsible for BTKi therapy, cardiologists for assessing and managing cardiovascular risks, and nephrologists for evaluating renal function and managing kidney-related complications [94].

Given the high bleeding rate associated with BTKi (most commonly ibrutinib in clinical practice), a thorough evaluation is necessary throughout the entire course of patient treatment. This entails gathering detailed information on the patient’s history of bleeding, concurrent medication usage, and the presence of any other conditions that elevate bleeding risk prior to treatment, as well as closely monitoring the patient’s bleeding status during the treatment [79]. Currently, an extensive management framework of “assessment-monitoring-intervention-restart” has been established [6]. Clinically, it is recommended to use second-generation BTKi with lower toxicity for patients at a high risk of bleeding, to minimize the risk of bleeding during treatment [54,104]. If bleeding events occur during treatment, appropriate management measures should be implemented based on the severity of the bleeding, including discontinuing or adjusting BTKi therapy, local hemostasis, platelet transfusion, administration of hemostatic drugs, surgical intervention, and other relevant measures [105,106]. A study using non-covalent BTKi to treat patients with hematological malignancies showed that the bleeding rate of patients is reduced. Once approved, it may represent the preferred alternative solution [79]. The bleeding mechanism of BTKi still needs to be fully explored, and relevant strategies should be provided to reduce the rate of bleeding and enable patients to receive better treatment.

**Fig. 3.**
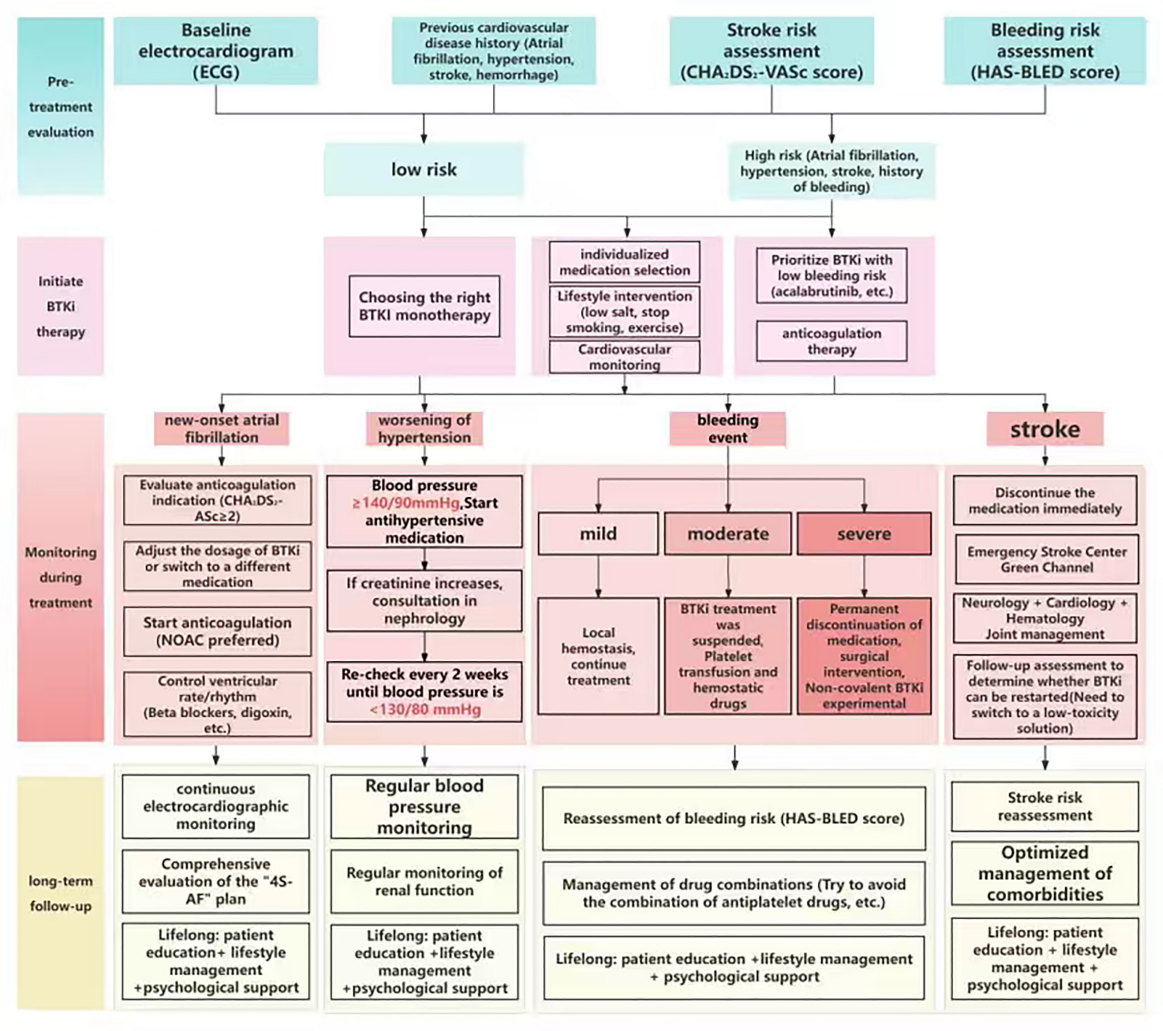
**Cardiovascular toxicity events associated with BTKis**. Before BTKi treatment, an assessment is required, which involves clinical management, evaluation using the CHA_2_DS_2_-VASc and HAS-BLED scales, and collection of medical histories such as AF and hypertension to assess the patient’s risk level. Patients at high risk are treated with low-bleeding-risk BTKi and anticoagulation therapy, while single-drug therapy is used for low-risk patients. Both patient groups are required to undergo cardiovascular monitoring and lifestyle intervention. During treatment, patients’ conditions are regularly monitored, and timely interventions are taken when abnormalities are detected. Long-term follow-up involves ambulatory ECG and blood pressure monitoring to assess bleeding and stroke risks, as well as long-term management of comorbidities, providing lifelong education and support to patients.

Although the incidence of stroke during BTK inhibitor treatment is relatively low, it still requires the attention of physicians to closely monitor cardiovascular events, especially the development of AF, in order to prevent the occurrence of stroke [107]. Oral anticoagulants remain the preferred treatment for stroke prevention in patients at increased risk of stroke [52,108]. Future research needs to further explore the differences between different BTKi and how to optimize treatment plans to reduce the occurrence of adverse cardiovascular events such as stroke [98]. As illustrated in Fig. 3, the clinical management workflow for BTKi-related cardiovascular toxicity events systematically summarizes the key steps of pre-treatment assessment, in-treatment monitoring, and long-term follow-up.

## 5. Future Research Directions

With the widespread use of BTKi in the treatment of B-cell malignancies, their significant anti-tumor efficacy has been fully validated in clinical practice, especially in the progression of diseases such as CLL and mantle cell lymphoma (MCL). However, the gradual manifestation of BTKi-related cardiovascular toxicity, especially AF, hypertension, bleeding, heart failure, and potential arrhythmias, has become an important obstacle limiting its widespread use and long-term management. Therefore, how to minimize cardiovascular adverse reactions while ensuring treatment efficacy has become a key issue that urgently needs to be addressed in the current clinical and scientific research fields.

Future research should explore the potential mechanisms of cardiovascular toxicity induced by BTKi from multiple perspectives, in order to provide a theoretical basis and practical guidance for the development and optimization of treatment strategies for novel low-toxicity BTKi. First, it is necessary to focus on the changes in key molecular pathways under the action of BTKi, especially the effects on the PI3K/Akt signaling pathway and ROS signaling pathway, as well as their potential off-target effects. The PI3K/Akt signaling pathway plays an important protective role in maintaining the survival and function of myocardial cells, while BTKi may inhibit this pathway, leading to programmed cell death or dysfunction of myocardial cells, thereby inducing heart failure or arrhythmias [109]. The excessive generation of ROS induced by BTKi promotes increased levels of oxidative stress, further exacerbating myocardial cell damage and inducing cardiovascular structural remodeling [110]. The off-target effect of BTKi also requires increased attention. In addition to inhibiting BTK, BTKi may also affect the activity of other kinases in the Tec family, leading to unexpected systemic toxicity [111]. This phenomenon is particularly significant in the cardiovascular system, which may lead to abnormal endothelial function, impaired cardiac electrical conduction, and platelet dysfunction. The effect of BTKi on platelets is also closely related to cardiovascular toxicity. BTKi has a broad inhibitory effect on BTK and Tec family kinases, which leads to abnormal platelet function. This not only increases the risk of bleeding, but may also indirectly exacerbate other cardiovascular complications. Therefore, developing more selective and targeted BTKi has become one of the key strategies to reduce its toxic side effects [112].

The development of new BTKi should not only focus on improving anti-tumor efficacy, but also aim to reduce cardiovascular toxicity as one of the key goals. Current research shows that although BTKi has significant therapeutic effects in the treatment of B-cell malignancies, the resulting CVAEs, especially AF, hypertension, and bleeding, significantly affect the patients’ quality of life and treatment compliance. Therefore, in the future design and development of BTKi, the focus should be on exploring in depth the following directions to reduce its risk of cardiovascular damage.

First, it is necessary to gain a deeper understanding of the molecular mechanisms underlying BTKi induced cardiovascular toxicity, including its interference with key signaling pathways such as PI3K/Akt, ROS, JAK/STAT, as well as the role of off-target inhibition of Tec family kinases in the cardiovascular system. This will provide mechanistic guidance for new drug development and promote the discovery of BTKi molecules that are more targeted and have higher cardiovascular safety. Second, in terms of drug structure optimization, efforts should be made to improve the selectivity and affinity of the binding between BTKi and BTK, and minimize non-specific inhibition of non-target kinases (such as ITK, TEC, etc.), thereby reducing toxic side effects such as cardiac electrical conduction disorders, vascular dysfunction, and platelet dysfunction. In addition, improving the bioavailability and pharmacokinetic properties of drugs can help reduce drug exposure doses while maintaining efficacy, thereby further reducing the burden on the cardiovascular system.

To achieve these goals, the development of the new BTKi urgently needs to be accelerated in multi-center clinical trials. Clinical trials should pay more attention to cardiovascular safety endpoints and risk stratification assessment, and strengthen monitoring of indicators such as electrocardiogram changes, blood pressure dynamics, and bleeding events during the treatment process. Although multi-center trials face challenges in data standardization, quality control, coordinated management, and funding, their high-quality data will provide strong support for the global application of BTKi and enhance control of cardiovascular toxicity.

The development of individualized treatment strategies is crucial for reducing BTKi-related cardiovascular risks. In the future, personalized risk prediction models can be established based on the patient’s genetic background, disease staging, past cardiovascular history, and comorbidities. On this basis, multiple omics data (such as genome, epigenome, transcriptome, etc.) can be further utilized to construct a precision medicine platform, predicting patients’ sensitivity, drug resistance, and cardiovascular adverse event risk to BTKi, thereby achieving truly personalized treatment and reducing toxic side-effects.

Firstly, it is essential to gain a deeper understanding of the molecular mechanisms underlying BTKi induced cardiovascular toxicity. This includes examining its interference with key signaling pathways such as PI3K/Akt, ROS, and JAK/STAT, as well as the role of off-target inhibition of Tec family kinases within the cardiovascular system. Such insights will provide valuable guidance for new drug development and facilitate the discovery of BTKi molecules that are more targeted and exhibit enhanced cardiovascular safety. Secondly, regarding drug structure optimization, efforts should focus on improving the selectivity and affinity of the binding between BTKi and BTK while minimizing non-specific inhibition of non-target kinases (such as ITK and TEC). This approach will help reduce toxic side effects, including cardiac electrical conduction disorders, vascular dysfunction, and platelet dysfunction. Additionally, enhancing the bioavailability and pharmacokinetic properties of these drugs can help lower exposure doses while maintaining efficacy, thereby further alleviating the burden on the cardiovascular system.

To achieve these objectives, it is crucial to expedite the advancement of multi-center clinical trials for new BTKis. These trials should emphasize the establishment of cardiovascular safety endpoints and risk stratification assessments, as well as strengthen monitoring of indicators such as electrocardiogram changes, blood pressure dynamics, and bleeding events throughout the treatment process. Although multi-center trials face challenges related to data standardization, quality control, coordinated management, and funding, their high-quality data output will provide robust support for the global application of BTKis and the management of cardiovascular toxicity.

Moreover, developing individualized treatment strategies is vital for mitigating BTKi-related cardiovascular risks. In the future, personalized risk prediction models could be established based on a patient’s genetic background, disease staging, past cardiovascular history, and comorbidities. Furthermore, various omics data (such as genomic, epigenomic, and transcriptomic information) can be utilized to construct a precision medicine platform that predicts patients’ sensitivity, drug resistance, and the risk of cardiovascular adverse events related to BTKi treatment. This will facilitate truly personalized treatment and targeted interventions.

## 6. Conclusion

In summary, to enhance the clinical application value of BTKis, future research should prioritize the identification of cardiovascular toxicity, mechanism analysis, and the development of systematic intervention strategies. Collaborative efforts across multiple levels are necessary, from drug design to clinical practice and personalized prediction and management, to achieve the dual goals of maximizing efficacy and optimizing safety.
